# Comparative Study of Proton Pump Inhibitors on Dexamethasone Plus Pylorus Ligation Induced Ulcer Model in Rats

**DOI:** 10.4103/0250-474X.70486

**Published:** 2010

**Authors:** A. H. M. Thippeswamy, M. Sajjan, M. B. Palkar, B. C. Koti, A. H. M. Viswanathaswamy

**Affiliations:** Department of Pharmacology, K. L. E. S College of Pharmacy, Vidyanagar, Hubli - 580 031, India

**Keywords:** Dexamethasone, PPIs, mucosal offensive and defense factors

## Abstract

The present study was designed to compare ulcer protective effect of proton pump inhibitors viz. omeprazole, rabeprazole and lansoprazole against dexamethasone plus pylorus ligation induced ulcer model. Dexamethasone (5 mg/kg) was used as an ulcerogen. Dexamethasone suspended in 1% CMC in water was given orally to all the rats 15 min after the pylorus ligation. Omeprazole (20 mg/kg), rabeprazole (20 mg/kg), and lansoprazole (20 mg/kg) were administered by oral route 30 min prior to ligation was used for ulcer protective studies, gastric secretion and mucosal studies. Effects of proton pump inhibitors were determined by the evaluation of various biochemical parameters such as ulcer index, free and total acidity, gastric pH, mucin, pepsin and total proteins. Oral administration of proton pump inhibitors showed significant reduction in gastric acid secretion and ulcer protective activity against dexamethasone plus pylorus ligation induced ulcer model. The % protection of omeprazole, rabeprazole and lansoprazole was 84.04, 89.36 and 79.78, respectively. Rabeprazole significantly inhibited the acid-pepsin secretion and increased the gastric mucin secretion. The observations made in the present study suggest that rabeprazole is the most effective gastric antisecretory and ulcer healing agent as compared to omeprazole and lansoprazole.

Acute gastric ulcers occur due to erosion in the mucosal membrane, generally in gastric and duodenum regions and occur as a result of alteration in the balance between mucosal damaging agents and mucosal defense mechanisms. Peptic ulcer is one of the most frequent disorders of the alimentary tract and in various countries its prevalence is estimated as 5-10% of the adult population[1]. This disorder remains one of the most important problems, both in the practice of primary health care physicians and gastroenterologists. It has been reported earlier that heavy smoking, alcohol and steroids intake may delay healing of ulcers. This could be due to increased gastric acid secretion, reduction in gastric mucosal blood flow, inhibition of duodenal bicarbonate production, prostaglandin synthesis, *Helicobacter pylori* infection, reduced generation of nitric oxide and increased generation of free radicals[2–5]. Ulcerogenic potential of corticosteroids is well known as a result of increased gastric acid and pepsin secretion, which will aggravate peptic ulcer[6]. Frequent usage of corticosteroids in the treatment of bronchial asthma, brain metastasis, cerebral edema etc has increased the risk of peptic ulcer[7]. Corticosteroids cause reduction in the levels of nitric oxide[8], inhibition of prostaglandin (PG) synthesis and formation of lipid peroxides[9] leading to gastric erosions by damaging surface epithelial cells.

Proton pump inhibitors (PPIs) inhibit release of hydrogen ion from parietal cells. It inhibits gastric acid secretion by blocking H^+^/K^+^ATPase pump. Omeprazole shows an ulcer healing effect by inhibiting neutrophil chemotaxis, superoxide production and release of active oxygen metabolites[10] leading to ulcer healing by augmenting luminal pH there by decreasing pepsin damage to gastric mucosa.

While lansoprazole prevents gastric mucosal damage by gastric prostaglandin production, expression of cyclo-oxygenase (COX) isoforms and release of stable nitric oxide metabolites into gastric juice and blocks the oxygen derived free radical output from neutrophils activated by *Helicobacter pylori* and exerts its antioxidant effect[11,12].

Rabeprazole causes perhaps the fastest acid suppression and so aid gastric mucin synthesis. This is necessary for the maintenance of mucosal integrity. Although these PPIs being similar in pharmacological actions they differ in clinical pharmacology[13]. Therefore, the present work was undertaken with an aim to compare different PPIs for the treatment of dexamethasone plus pylorus ligation induced ulcer model in albino rats.

Omeprazole was obtained from Cipla Ltd, Goa, India. Rabeprazole, lansoprazole and dexamethasone were obtained from Cadila health care, Ahmedabad, India. The chemicals and solvents used were sodium hydroxide, Topfer’s reagent, copper sulphate, phenolphthalein, sodium carbonate, phenol reagent, bovine albumin, sucrose, alcian blue, sodium acetate, ethanol, methanol, dil. HCl and chloroform. All were of analytical grade.

Healthy Wistar rats of either sex weighing between 150-200 g were used. Animals were housed individually in polypropylene cages, maintained under standard conditions, (12:12 L:D cycle; 25±3°and 35-60% humidity) the animals were fed with standard rat pellet diet, (Hindustan Lever Ltd., Mumbai, India) and water *ad. libitum*. The study was conducted after obtaining institutional animal ethical committee clearance.

Dexamethasone (5 mg/kg) suspended in 1% w/v carboxy methyl cellulose (CMC) in water was given orally to all the rats 15 min after the pylorus ligation. Omeprazole (20 mg/kg), rabeprazole (20 mg/kg), and lansoprazole (20 mg/kg) were administered by oral route, 30 min prior to ligation was used for ulcer protective studies, gastric secretion and mucosal studies.

Wistar rats of either sex were divided into four groups of 6 animals each. In this method rats were fasted in individual cages for 24 h. Care was taken to avoid coprophagy. Control vehicle, omeprazole (20 mg/kg), rabeprazole (20 mg/kg) and lansoprazole (20 mg/kg) were administered by oral route 30 min prior to ligation. Dexamethasone suspended in 1% w/v CMC was given orally to all the rats 15 min after pylorus ligation. Under ether anesthesia, the abdomen was opened and the pylorus was ligated[14]. The abdomen was then sutured and animals were allowed to recover from the anesthesia. The animals were sacrificed after 4 h, stomachs were dissected out and gastric contents were drained into tubes and subjected to estimation for pH, free and total acidity[15], total proteins[16], pepsin[17] and glandular portions for mucin[18]. The stomachs were then cut open along the greater curvature and the severity of hemorrhagic erosions in the acid secreting mucosa was assessed on a scale of 0 to 3 and ulcer index was determined. Based on their intensity, the ulcers were given scores as follows; 0 indicates no ulcer, 1 denotes superficial mucosal erosion, 2 indicates deep ulcer or transmural necrosis and 3 denotes perforated or penetrated ulcer. The ulcer index was determined using the formula[19], Ulcer index= 10/X, where X is the total mucosal area/total ulcerated area. The results obtained from the present study were analyzed using one-way ANOVA followed by Dunnett’s multiple comparison test using GraphPad Prism 5. The results were expressed as the mean±SEM.

The PPIs significantly reduced the gastric volume, total and free acidity, and increased the pH of the gastric fluid, proving its antisecretory activity. Animals in the omeprazole, rabeprazole and lansoprazole groups showed decrease in volume of gastric juice by 41.17, 48.46 and 34.31 %, respectively, free acidity was found to be 51.13, 63.44 and 30.60 %, respectively and total acidity was found to be 47.65, 55.16 and 35.07 %, respectively. pH was found to increase by 148.87, 166.85 and 134.83 %, respectively. It is evident from the results that rabeprazole is more effective than omeprazole and lansoprazole (Table 1).

**TABLE 1:** EFFECT OF PROTON PUMP INHIBITORS ON VOLUME OF GASTRIC JUICE, pH, FREE ACIDITY AND TOTAL ACIDITY

Group	Treatment	Volume of gastric juice (ml)	pH	Free acidity (Meq/100 g)	Total acidity (Meq/100 g)
I	Control	11.90±0.11	1. 78±0. 09	44.6 7±0.49	88.83±0.87
II	Omeprazole	7.00±0.27^a^	4.43±0.1 0^a^	21.83±0.95^a^	46.50±2.26^a^
III	Rabeprazole	6.13±0.27^a^	4.75±0.09^a^	16.33±1.26^a^	39.83±1.54^a^
IV	Lansoprazole	7.82±0.22^a^	4.18±0.16^a^	31.00±1.37^a^	57.67±2.47^a^

Values are mean±SEM; n=6.

a *p*<0.01 when compared to control group.

Percent protection in ulcer index offered by omeprazole, rabeprazole and lansoprazole was 83.92, 89.28, and 79.45, respectively (Table 2). Rabeprazole was found to be most effective. There was significant increase in mucin and reduction in protein and pepsin content in PPIs treated groups compared to control group (Table 3).

**TABLE 2:** EFFECT OF PROTON PUMP INHIBITORS ON ULCER INDEX

Group	Treatment	Ulcer index	Protection (%)
I	Control	9.33±0.25	---
II	Omeprazole	1.50±0.22^a^	83.92
III	Rabeprazole	1.00±0.20^a^	89.28
IV	Lansoprazole	1.92±0.37^a^	79.45

Values are mean±SEM; n=6.

a *p*<0.01 when compared to control group.

**TABLE 3:** EFFECT OF PROTON PUMP INHIBITORS ON MUCIN, TOTAL PROTEINS AND PEPSIN

Group	Treatment	Mucin content (μg/g of wet gland)	Total proteins (μg/ml)	Pepsin (μg/ml)
I	Control	216.7±1.453	355.3±1.856	15.02±0.691
II	Omeprazole	242.3±3.266^a^	309.2±5.974^a^	7.5±0.1706^a^
III	Rabeprazole	255.8±5.608^a^	280.0±3.941^a^	6.6±0.5258^a^
IV	Lansoprazole	236.8±4.400^b^	321.7±6.540^a^	8.8±0.2352^a^

Values are mean±SEM; n=6.

a *p*<0.001 when compared to control group.

b *p*<0.01 when compared to control group.

In the present study gastric ulcer was induced by dexamethasone plus pylorus ligation model. Pylorus ligation induced ulcers are due to autodigestion of the gastric mucosa and break down of the gastric mucosal barrier. Reactive oxygen species are involved in the pathogenesis of pylorus ligation induced mucosal injury[20]. Gastric ulcer and gastrointestinal bleeding are recognized complications of corticosteroid therapy. It causes ulcers by increase in the generation of lipid peroxides indicating the involvement of free radicals in the process of ulceration[21]. Ulcerogenic potential of corticosteroids is well known and is believed to be a result of increased gastric acid secretion and decrease in PGs synthesis. It also delays the gastric ulcer healing by inhibiting epithelial cell proliferation and angiogenesis at the ulcer margin. In dexamethasone plus pylorus ligation model, PPIs viz. omeprazole, rabeprazole and lansoprazole significantly decrease the gastric juice volume, acid and pepsin output indicating decrease in offensive acid and pepsin secretion. On the defensive factors, PPIs significantly increased the gastric mucin secretion and prevented the gastric mucosal damage induced by dexamethasone plus pylorus ligation. Among these PPIs, rabeprazole showed better reduction of gastric acid secretion and decrease in ulcer index than to omeprazole and lansoprazole. This effect of rabeprazole may be due to rapid onset of H^+^/K^+^ ATPase pump inhibition and a greater effect on intragastric pH as compared to omeprazole and lansoprazole. The present findings agree with earlier reports.

Omeprazole, rabeprazole and lansoprazole have shown increased gastric pH and decrease in the protein content of gastric juice. Gastric acid is an important factor for the genesis of ulceration of pylorus ligation ulcer in rats. Gastric acid secretion is influenced by many factors including anxietic effect in the CNS, vagal activity, cholinergic, histaminergic and gastrinergic transmissions[22]. The antisecretory actions of PPIs mainly involve inhibition of H^+^/K^+^ ATPase pump. Cytoprotective effect of omeprazole is due to increased expression of COX-2 protein and elevating the levels of PGE_2_. It also showed increased gastric pH and reduction in gastric acid secretion, which may be due to inhibition of gastric mucosa enzymes, carbonic anhydrase II (CA) and CA IV, which are located in abundance in the gastric parietal cells and in the secretory canaliculi walls. This inhibition potentiates the inhibitory effect on the proton pump[23]. Similarly lansoprazole exerts its gastroprotective effects by increased bio-availability of mucosal sulfhydryl compounds and possibly prostaglandins[24].

Amount of gastric mucus secretion in gastric mucosa was found to be increased in rabeprazole treated group[25]. This mucus consists of mucin-type glycoproteins, which can be detected by amount of alcian blue binding[26] (fig. 1). The decrease in the protein content of gastric juice by rabeprazole suggests the decrease of leakage of plasma proteins into gastric juice. This further suggests the increase in glycoprotein content of the gastric mucosa and that acts as a coating as well as protective barrier on the mucosa.

**Fig. 1:**
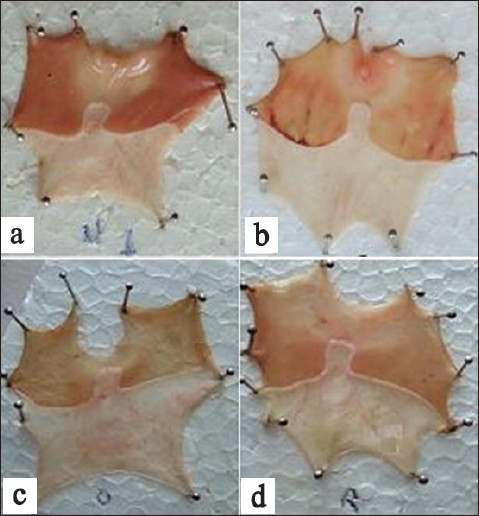
Ulcer index in dexamethasone plus pylorus ligated induced ulcer model (a) Control, (b) omeprazole treated group, (c) rabeprazole treated group and (d) lansoprazole treated group

Thus, the ulcer healing effect of rabeprazole may be due to its effect on both offensive and defensive factors. Further work on other mucosal factors like nitric oxide, prostaglandins, cAMP etc would provide more insight into the activity of rabeprazole.
